# Out of the Pacific and Back Again: Insights into the Matrilineal History of Pacific Killer Whale Ecotypes

**DOI:** 10.1371/journal.pone.0024980

**Published:** 2011-09-20

**Authors:** Andrew D. Foote, Phillip A. Morin, John W. Durban, Eske Willerslev, Ludovic Orlando, M. Thomas P. Gilbert

**Affiliations:** 1 Centre for GeoGenetics, Natural History Museum of Denmark, University of Copenhagen, Copenhagen, Denmark; 2 Protected Resources Division, Southwest Fisheries Science Center, National Marine Fisheries Service, National Oceanic and Atmospheric Administration, La Jolla, California, United States of America; 3 Alaska Fisheries Science Center, National Marine Fisheries Service, National Oceanic and Atmospheric Administration, Seattle, Washington, United States of America; University of Texas Arlington, United States of America

## Abstract

Killer whales (*Orcinus orca*) are the most widely distributed marine mammals and have radiated to occupy a range of ecological niches. Disparate sympatric types are found in the North Atlantic, Antarctic and North Pacific oceans, however, little is known about the underlying mechanisms driving divergence. Previous phylogeographic analysis using complete mitogenomes yielded a bifurcating tree of clades corresponding to described ecotypes. However, there was low support at two nodes at which two Pacific and two Atlantic clades diverged. Here we apply further phylogenetic and coalescent analyses to partitioned mitochondrial genome sequences to better resolve the pattern of past radiations in this species. Our phylogenetic reconstructions indicate that in the North Pacific, sympatry between the maternal lineages that make up each ecotype arises from secondary contact. Both the phylogenetic reconstructions and a clinal decrease in diversity suggest a North Pacific to North Atlantic founding event, and the later return of killer whales to the North Pacific. Therefore, ecological divergence could have occurred during the allopatric phase through drift or selection and/or may have either commenced or have been consolidated upon secondary contact due to resource competition. The estimated timing of bidirectional migration between the North Pacific and North Atlantic coincided with the previous inter-glacial when the leakage of fauna from the Indo-Pacific into the Atlantic via the Agulhas current was particularly vigorous.

## Introduction

The classical interpretation of the theory of speciation suggests that an allopatric phase of two or more populations of the ancestral species is essential to prevent gene flow and achieve reproductive isolation [1], [2]. Divergence and reproductive isolation can then be consolidated if the two divergent forms come into secondary contact, for example through reinforcement, e.g. resource competition leading to selection for extreme phenotypes and reduced hybrid fitness [3], [4]. However, recent theoretical models have demonstrated potential mechanisms for sympatric speciation [5]–[8], and several empirical studies have argued that such mechanisms do operate in nature [9]–[12]. Sympatric speciation is likely to require strong natural selection on traits that are heritable and which are also linked to mate choice to overcome the homogenizing effect of gene flow [5]–[8]. Identifying the origins and subsequent dispersal and migrations of taxa can help elucidate the geographic context and underlying mechanisms and processes responsible for population structure and/or speciation (e.g. [13]–[15]). For example, did divergence take place during sympatry, or did sympatry arise from secondary contact; was divergence vicariant or with low levels of on-going gene flow; what was the relative importance of drift and selection (e.g. [13], [14], [16]). Applying phylogenetic and coalescent analyses to address such questions can provide insights into the species' geographic origins, historic radiations, and the mode of speciation (e.g. [13], [16]–[20]).

The killer whale (*Orcinus orca*) is a useful model species with which to investigate the spatial and temporal context of genetic divergences, as both geographic separation and ecological specialisation appear to be drivers of population structure [21]–[24]. In this study we aim to resolve the outstanding question of the mode of evolutionary and ecological divergence of three killer whale ecotypes that currently occur in partial sympatry in the eastern North Pacific.

Although currently considered a single species, the killer whale has radiated into morphologically and ecologically disparate types in the Antarctic, North Atlantic and North Pacific [25]–[29]. A previous study indicated that the worldwide pattern of mitochondrial DNA (mtDNA) diversity for this species was consistent with a historical bottleneck, followed by a rapid radiation leading to a stochastic geographic distribution of lineages [29]. A number of analyses were presented which supported this hypothesis; mtDNA haplotypes were shared between ocean basins, the phylogeny was star-shaped and mismatch distribution was unimodal, and variation at five nuclear microsatellite loci was also low [29]. However, the phylogenetic analyses used were limited to the mitochondrial control region (995 base pairs), which represents only 6% of the killer whale mitochondrial genome [23]. Many sites within this often hyper-variable region of the mammalian mtDNA are believed to be mutational hot spots [30], [31] resulting in a loss of phylogenetic resolution [19]. Given these limitations, a larger phylogenetic analysis [23] that used complete mitogenomes was subsequently performed which demonstrated almost complete lineage sorting of all killer whale types, and dated divergence times of several major clades to prior to the date of the bottleneck suggested by Hoelzel et al. [29].

The reconstructed mitogenome phylogeny indicated that three Antarctic lineages (AntA1, AntA3, AntA4), all classified as type A (see [26]), and three North Atlantic lineages (ENACI2, WNAGM, ENA2S2) are sister taxa and cluster together in a paraphyletic clade with the other Antarctic lineages [24]. This suggests a more recent movement of these lineages from the Antarctic into the North Atlantic, resulting in secondary contact between North Atlantic clades [24]. The three Antarctic types for which mitogenomes were sequenced (types A, B & C) are sister taxa [23], suggesting that evolutionary divergence occurred in Antarctica and potentially in the face of ongoing gene flow. However, studies with strong evidence for sympatric speciation are typically those in which the geographic context is small and isolated enough to rule out any allopatric phase, e.g. fish in crater lakes or birds and plants on small remote oceanic islands [10]–[11]. Past glaciations and the strong ocean temperature gradient across the polar frontal zone that separates the Antarctic and sub-Antarctic are potential barriers that could have resulted in the Antarctic types being temporarily isolated from one another in localised refugia [32]. However, these are arguably the most morphologically divergent of killer whale types [22], [33], and the only types for which natural selection on heritable traits has been demonstrated to date [34]. Stronger selection may therefore have driven phenotypic divergence despite the potential for on-going low-levels of gene flow between Antarctic types.

There was a relatively large phylogenetic divergence between the North Pacific ‘transient’ type and the other two North Pacific types (‘resident’ and ‘offshore’), which were clustered with killer whales sampled in the North Atlantic. This could imply that sympatry with the transient type arose from secondary contact. However, the chronology of divergences amongst the North Atlantic and North Pacific offshore and resident clades was not clearly resolved, and there was low support (posterior probabilities of 0.299 and 0.533) for divergences at the two nodes of these clades [23]. Therefore, the sequential radiation of currently delineated killer whale types has not yet been fully resolved.

In this study we apply novel analyses to a previously published dataset of killer whale mitogenome sequences to further investigate if the North Pacific ecotypes diverged in the Pacific and there was subsequent founding of the Atlantic populations, or if sympatry of the North Pacific ecotypes arises from secondary contact following an allopatric phase during which the ancestors of the resident and offshore lineages were in the Atlantic.

## Materials and Methods

Individuals were assigned to type before sequencing based upon morphological or behavioural data (see [23], [34]). The North Pacific transient type is a mammal-eating specialist, the Pacific resident type is a fish-eating specialist, and the Pacific offshore type is also thought to be piscivorous, with sharks appearing to be a key component of the diet [24], [35]–[39]. There are subtle morphological differences among the North Pacific types [25], [40], which have been confirmed by molecular analysis to be reliable in determining type in the absence of predation observations [41]. When assignment of an individual to type was not possible prior to sequencing, the individual was classified as type unknown.

### Phylogenetic analyses

Firstly, we aimed to improve phylogenetic resolution and increase the support at the nodes at which clades 2–5 diverge by using partitioned data, which can improve phylogenetic resolution (e.g. [42]). We used the mitogenome sequences of 143 individuals, which have been published by two previous studies [23], [24] (see Table S1 for accession numbers). This included 135 complete and 8 partial mitochondrial genome sequences, and the long-finned pilot whale, *Globicephala melas*, as an outgroup sequence [23]. Sequences were aligned and subsequently pruned to retain only the 2 rRNA and 13 protein-encoding genes (ca. 14,038 bp in length). The control region was excluded from analysis due to suspected saturation and repetitive sequences. The resulting alignment was divided into four partitions: the first, second and third codon sites of the protein- coding genes (3,827 bp per partition) and the rRNA genes (2,557 bp). The most likely model of evolution that was compatible with models implemented in MrBayes 3.1.2 [43] was selected for each partition using jModelTest 1.1 [44] using the corrected Akaike Information Criterion (AIC). The selected models were GTR+G for the first codon position of the protein-coding genes and HKY for the second and third codon positions and the rRNA genes. Four independent Monte Carlo Markov chains (MCMC) were run simultaneously for 1,000,000 generations with the current tree and parameter values sampled every 100 generations. Convergence was judged to have occurred when the standard deviation of split frequencies across the chains was <0.01 after 1,000,000 generations resulting in 10,000 trees, of which the first 25% (2,500) were discarded as burn-in. The potential scale reduction factor (PSRF) was 1.0 for all parameters. The majority-rule consensus tree was summarised from the final 7,500 trees, and support for clades was expressed as posterior probabilities.

Secondly, we applied an additional approximately unbiased test of phylogenetic tree selection to further investigate the most likely branching order of clades 1–5. Maximum-likelihood phylogenetic analysis of the non-partitioned protein-coding and rRNA gene sequences (14038 bp) was performed, using PhyML 3.0 [45], using the best-fit model of nucleotide substitution of HKY for the non-partitioned data, selected as above. The transition/transversion ratio, the proportion of invariable sites, and the gamma distribution were estimated using PhyML. The starting tree was estimated using BIONJ algorithm implemented in PhyML. The site-wise likelihood values recovered from the PhyML analysis were subsequently used to evaluate the levels of statistical support for alternative topologies, each consistent with a different chronology of divergences. Statistical support for each topology was evaluated from the *P*-values of approximately unbiased (AU) tests [46] estimated using Consel 0.1 k [47]. The AU test estimates the probability that the tree is the true tree by calculating the approximately un-biased *P*-values from the change in the bootstrap probability values along the changing sequence length [46]. Trees can be rejected as representing the true tree at a significance level *α*<0.05 [46].

### Genetic diversity measures

Step-wise founding events are expected to lead to a concurrent step-wise reduction in genetic diversity with each subsequent founding event (e.g. [48]), though this will also be influenced by demographic factors such as subsequent effective population size and immigration. We used DnaSP v5 [49] to calculate a number of genetic diversity measures for each clade with at least 4 complete sequences: the number of segregating sites (*S*), the number of segregating sites per nucleotide site (*P_S_*), haplotype diversity (*θ*_h_), nucleotide diversity (*π*) and Tajima's *D* [50]. We use the sub-notation ‘h’ to differentiate between haplotype diversity (*θ*_h_) and Watterson's theta (*θ*), which is an estimate of population mutation rate and is a parameter in the isolation with migration analysis below.

### Isolation with migration analysis

To further investigate the temporal pattern of migration between ocean basins we estimated population sizes, the time of splitting, and migration rates by implementing the ‘isolation with migration’ (IM) model of population divergence [51] using IMa [52], run on the BioHPC clusters at Cornell University. Initial runs without data (-j0) were used to assess the boundaries of the assumed prior distributions for parameters and to ensure that the posterior distributions fell within the prior range. Non-partitioned sequences of the protein-coding and rRNA genes were used in the input file. The substitution rate per year for the locus (4×10^−5^) was estimated by multiplying the per-site values for the complete mitogenome in Morin et al. [23] by 14038. A published generation time of 15 years estimated from long-term photo-identification studies [53] was used. Five independent replications of 100,000,000 iterations, with a 10% burn-in period under a finite sites model (HKY) were performed and then combined in an L mode analysis. To ensure convergence, the five independent runs were compared for repeatability, the mixing properties of the MCMC were assessed by visual inspection of the parameter trend plots and by examining the level of autocorrelation between initial and final parameter values using the effective sample size (ESS), and ensuring that update rates were greater than 20%. Convergence upon the stationary distribution was accepted if independent runs generated similar posterior distributions, with each having ESS of at least 50 for each parameter as recommended by Hey & Nielsen [51]. The posterior estimates of the model were scaled into demographic units using the locus mutation rate per generation (μ, based upon the per year estimate above and assuming a generation time of 15 years). The population mutation rates (Watterson's theta; *θ*_1_, *θ*_2_ and *θ*_A_) were converted into estimates of effective population size parameters (*N*_1_, *N*_2_ and *N_3_*) where *N*_e_ = θ/2μ, time since splitting (*t*) was converted to calendar years (*t* = τ/μ), and directional migration rates (*m*_1_, *m*_2_) were converted to the effective number of female migrants per generation (*M* = *m*μ).

## Results and Discussion

The results of this study provide further resolution of the chronology of phylogenetic divergence and the movement between ocean basins of killer whale types, providing new insights into the geographic context and potential mode of speciation. In particular, these new analyses strongly suggest an allopatric phase and secondary contact of the partially sympatric Pacific ecotypes. The ancestral maternal lineages of the Pacific resident and offshore ecotypes appear to have migrated back to the Pacific from the Atlantic.

### Phylogenetic analyses

**Figure 1:**
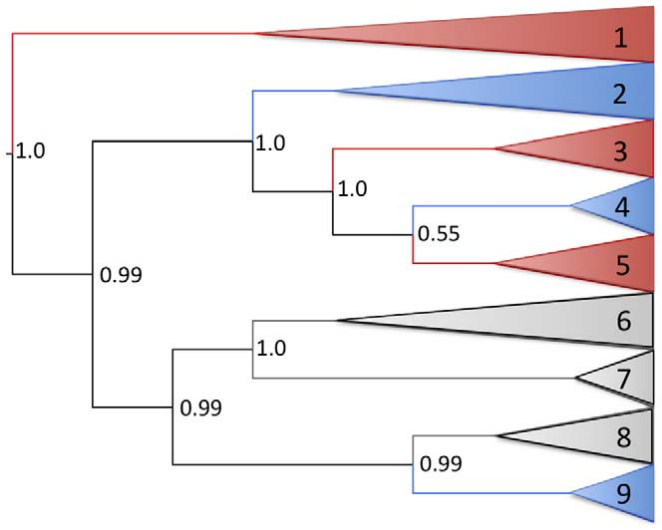
Bayesian partitioned mitogenome phylogeny. Clades are collapsed. Branch colours indicate geographic origin of samples as follows: Antarctic (grey), Atlantic (blue) and Pacific (red). The predominant ecotype or type in each clade is as follows: clade 1: Pacific transient; clade 2: Atlantic haplotypes from the Icelandic and Norwegian herring-eating populations and Gibraltar tuna-eating population; clade 3: Pacific resident; clade 4: Atlantic haplotypes from UK, Canary Islands and Iceland; clade 5: Pacific offshore; clade 6: Antarctic type C; clade 7: Antarctic type B; clade 8: Antarctic type A; clade 9: Atlantic haplotypes from Scotland, Canary Island and Gulf of Mexico. Posterior probabilities are given for nodes of interest. The tree is rooted with long-finned pilot whale (not shown).

Phylogenetic estimates were consistent across both Bayesian analysis of the partitioned sequence data (Fig. 1, Fig. S1) and maximum-likelihood analysis of the unpartitioned sequence data (not shown), and with those proposed in previous studies [23], [24]. The analysis of the partitioned data resulted in higher support at the two nodes that previously had low support [23], [24], however, the support for the node at the divergence of clades 4 and 5 was still relatively low (0.55).

**Figure 2:**
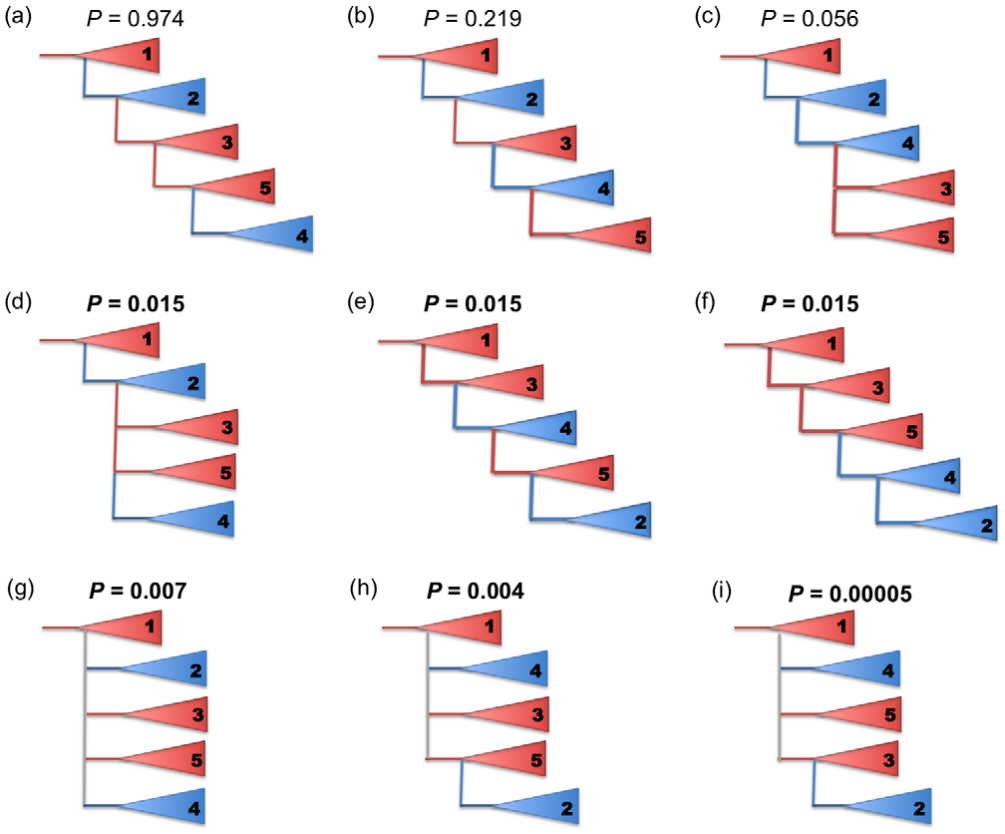
Topologies compared and ranked using an approximately unbiased test of tree selection. Topologies are presented in order of ranking and *P*-values are shown. The branching order of the only strongly supported (*P* = 0.974) topology suggests bidirectional movement between the Atlantic (blue) and the Pacific (red). Topologies that have significant *P*-values at *α*≤0.05 (shown in bold) can be rejected. This included all those that were not consistent with an allopatric period for the Pacific offshore and resident ecotypes.

The only topology with strongly significant support (*P* = 0.974) from the approximately unbiased test run using Consel 0.1 k was consistent with a series of dispersal events rather than vicariant splits (Fig. 2). The results strongly imply that the partial sympatry between the North Pacific transient, resident and offshore lineages arises out of secondary contact following the migration of the resident and offshore lineages from the North Atlantic to the North Pacific. Migration appears to have been bidirectional as a second North Atlantic clade is nested within the North Pacific clade containing the offshores. We could reject with a level of significance of *α*≤0.05 all topologies not consistent with the resident and offshore clades having originated from the North Atlantic. There were two alternative topologies that we were unable to significantly reject, but which had very low support (*P* = 0.219 and *P* = 0.056). These differed in the order of dispersal of the North Pacific offshore clade and the second North Atlantic clade (Fig. 2). Divergences among these clades are estimated to have occurred relatively rapidly approximately 200 (80–300 95% HPDI) KYA [23]. Such rapid divergences would explain the uncertainty over the chronology of dispersal and the relatively low support (0.55) at the node between the mixed clades 4 and 5.

### Genetic diversity measures

Genetic diversity summary statistics *θ* and *π* were also consistent with the inferred chronology of founding events from a North Pacific source population to the North Atlantic and then back to the North Pacific (Table 1). However, these results need to be interpreted cautiously as demographic expansion and selection can also influence these values. Values for Tajima's *D* were non-significant (*P*>0.1) and therefore not indicative of a strong departure from mutation-drift equilibrium. It should be noted that *θ*_h_ assumes that mutations are neutral, and samples are drawn from a randomly mating population that has remained constant in size. If *θ*_h_ tends to be greater than *π*, as is the case here, then this can indicate that the assumptions required for estimating *θ*_h_ are not met. The fact that the samples within each clade were not drawn from a single randomly mating population would make this highly likely. However, *θ*_h_ and *π* are highly correlated (*r* ^2^ = 0.872) within our dataset and *π* is not subject to the same assumptions. Therefore the difference in diversity summary statistics between clades does not appear to be simply an artefact of an undetected departure from mutation-drift equilibrium.

**Table 1:** Summary genetic diversity statistics for clades containing four or more complete mitogenome sequences.

Ocean Basin	Clade	Type	n	S	P_S_	θ_h_	π	D
Pacific	1	Transient	24	28	2.0×10^−3^	5.4×10^−4^	4.7×10^−4^	−0.49
Atlantic	2	Type 1	21	25	1.8×10^−3^	5.0×10^−4^	3.5×10^−4^	−0.56
Pacific	3	Resident	15	18	1.3×10^−3^	4.0×10^−4^	2.1×10^−4^	−1.29
Pacific	5	Offshore	16	11	0.8×10^−3^	2.4×10^−4^	2.0×10^−4^	−0.51

The statistics are number of segregating sites (*S*), the number of segregating sites per nucleotide site (*P_S_*), haplotype diversity (*θ*_h_), nucleotide diversity (*π*) and Tajima's *D*.

Nucleotide diversity (*π*) was relatively low and Tajima's *D* statistic was negative for all clades in Table 1, which although non-significant, could still suggest a historic population expansion. Hoelzel et al. [29] suggested a bottleneck event *ca*. 150–210 KYA. In addition to the estimated time to most recent common ancestor (TMRCA) of the two North Atlantic clades and the North Pacific resident and offshore clades being approximately 200 KYA, Morin et al. [23] also estimated the TMRCA for the North Pacific transients at 0.19 (0.10–0.31 95% HPDI) MYA. A bottleneck for several clades during the glacial maximum at approximately 200 KYA [54] does therefore appear to be a potential cause of reduced genetic diversity at both mtDNA and nuclear loci [29]. However, the analysis of the mitogenome sequences differed from analysis of the mtDNA control region [29], as it suggested that many of the known killer whale ecotypes had diverged prior to this glacial maximum [23]. A historical bottleneck at approximately 200 KYA would have accelerated coalescence and lineage sorting of the clades that had already diverged at this point in time [55]. This would also further confound interpretation of diversity measures as indicators of the chronology of divergences.

### Isolation with migration analysis

The independent replicated IMa runs provided consistently similar posterior probability distributions for all parameters and displayed good mixing properties of the MCMC chains as indicated by the parameter trend line plots and ESS values of greater than 50. Although the mitochondrial genome is a single locus, the analyses produced clear curves with consistent posterior distributions, suggesting good resolution of the 6 parameters being estimated.

The results also suggest migration of maternal lineages has been bi-directional between the North Pacific and North Atlantic, but at a very low level; the number of effective migrants per 1,000 generations (15,000 years) was estimated at 0.058 (0.003–0.129 90% Highest Probability Density Interval, HPDI) into the North Atlantic, and 0.043 (0.003–0.099 90% HPDI) into the North Pacific. Based on this estimate the number of founding females of the resident and offshore lineages would be very small, potentially even a single female. The establishment and persistence of reproductively isolated and evolutionary divergent populations from a single migrant following secondary contact have been noted in other species e.g. Darwin's finches (*Geospiza fortis*) [56]. Such case studies highlight how stochastic events can play a key role in speciation [56]. The estimates of the number of effective females, (*N*_Atlantic_ = 68,942, 90% HPDI = 33,564–103,144; *N*_Pacific_ = 66,608, 90% HPDI = 41,404–90,894), appeared large, but not unrealistic, given that abundance estimates from line transect surveys have been in the tens of thousands for waters around Iceland in the North Atlantic [57], and in the thousands for a relatively small coastal area around the Aleutian chain in the North Pacific [41]. Migration from un-sampled ‘ghost’ populations can also moderately inflate estimated effective population size [52]. As the ancestors of North Atlantic lineages within clade 6 (Type 2 and Mid-Atlantic) have clearly migrated from the Antarctic, the estimated *N*_e_ of the ancestral female population may therefore be more representative of the female *N*_e_ for the genus as a whole. The estimated time of splitting between killer whale lineages in the North Pacific and North Atlantic was 630 (270–1000 90% HPDI) KYA, which is consistent with phylogenetic estimates of the time since splitting of all other killer whales from the North Pacific transients of 702 (489–956 95% HPDI) KYA [23]. This would be consistent with an initial radiation during the inter-glacial that commenced approximately 650 KYA [58].

**Figure 3:**
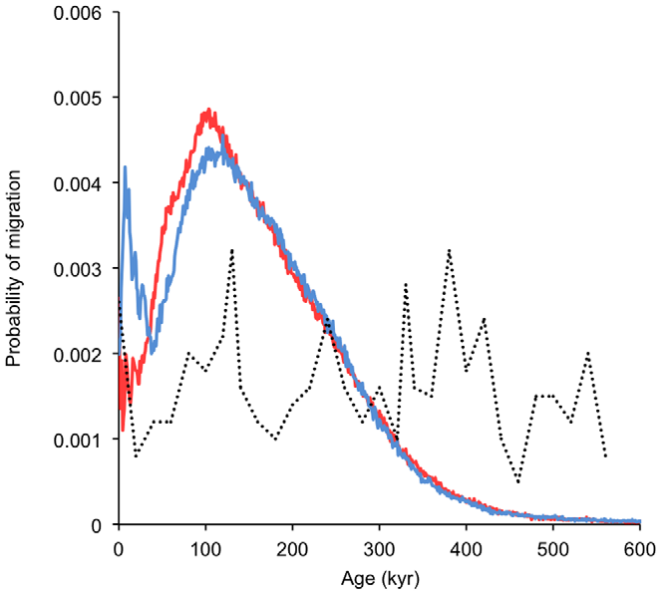
Probability distributions for migration events through time into the Atlantic (blue line) and Pacific (red line). The black broken line indicates the relative abundance of Agulhas leakage fauna based on data presented in ref. [59].

The IMa analyses suggested that migration events of females between the North Atlantic and North Pacific have taken place in both directions (Fig. 3). There is an overlap in the peak of the probability density distributions for migration events in each direction (Fig. 3), which occurs during the previous inter-glacial period (approximately 131–114 KYA BP [54]). This peak coincides with a period of maximal ‘Agulhas leakage’: the vigorous exchange of fauna that occurred between the Indo-Pacific and the southwest Atlantic during the inter-glacials of the late Pleistocene, promoted by an enhanced Agulhas current around the southern tip of Africa [59]. Fish stocks thought to have colonised the North Atlantic and Mediterranean during these episodic oceanic inter-changes include other top marine predators such as the great white shark (*Carcharodon carcharias*) [20] and species predated by killer whales such as bluefin tuna (*Thunnus thynnus*) and swordfish (*Xiphias gladias*) [60]. This could therefore have been the driver of a period of rapid episodic inter-oceanic interchange of killer whale lineages. A secondary more recent peak of migration into the North Atlantic was inferred to have occurred at the start of the current inter-glacial, which started approximately 11 KYA BP [54] (Fig. 3). Inspection of the phylogenies in the Figure S1 and reference [23] suggests this secondary peak is probably inferred from the inclusion of a sequence of an individual sampled in the Atlantic (Newfoundland Canada) that had a haplotype (WNAUCAN) nested within the North Pacific offshore haplotypes in clade 5.

### Insights into the mode of divergence

Although ecologically and sometimes morphologically disparate types of killer whale currently exist in partial sympatry in the Antarctic, Atlantic and Pacific, in at least two of these ocean basins (the North Pacific and North Atlantic) this appears to result from secondary contact rather than sympatric divergence. An isolated allopatric phase would allow the accumulation of mutations through drift and/or selection leading to genetic, ecological and phenotype divergence between isolated populations. Genetic divergence could lead to reproductive isolation through the epistatic interactions, including Dobzhansky-Muller incompatibilities between mutations accumulated in different populations [61]. However, incompatibilities could have arisen, or have been further promoted upon secondary contact through resource competition promoting specialisation and further divergence [3], [4].

The new analyses of partitioned mitochondrial genome sequences presented here provide further resolution of the matrilineal history of killer whale types. However, mtDNA may not fully reflect the underlying pattern of divergence and lineage formation if there is male mediated gene flow between ecotypes upon secondary contact. Previous results based on nuclear data (microsatellites) found a mid-Atlantic population containing individuals from all three Atlantic clades, indicating that secondary contact in killer whales does not always promote reproductive isolation [24]. Microsatellite analyses also suggest low levels of gene flow between Pacific killer whale ecotypes [22]. Therefore, future work should focus on comparing nuclear gene allele frequencies among populations and types.

## Supporting Information

Figure S1 **Bayesian phylogeny of the samples analysed showing internal nodes of each clade.** Branch colours indicate geographic origin of samples as follows: Antarctic (black), Atlantic (blue) and Pacific (red). Bold numbers indicate the basal node to the clades as used in the AU test and genetic diversity summary statistics. Posterior probabilities are given for nodes of interest. The tree is rooted with long-finned pilot whale (not shown).
(TIFF)

Table S1 List of GenBank accession numbers of sequences used in this study.
(DOC)

## References

[1] Dobzhansky T (1937). Genetics and the Origin of Species.

[2] Mayr E (1942). Systematics and the Origin of Species.

[3] Schluter D (2000). Ecological character displacement in adaptive radiation.. Am Nat.

[4] Servedio MR, Noor MAF (2003). The role of reinforcement in speciation: theory and data.. Annu Rev Ecol Evol Syst.

[5] Dieckmann U, Doebeli M (1999). On the origin of species by sympatric speciation.. Nature.

[6] Doebeli M, Dieckmann U (2003). Speciation along environmental gradients.. Nature.

[7] Bolnick DI, Fitzpatrick BM (2007). Sympatric speciation: models and empirical evidence.. Annu Rev Ecol Evol Syst.

[8] Nosil P (2008). Speciation with gene flow could be common.. Mol Ecol.

[9] Barluenga M, Stolting KN, Salzburger W, Muschick M, Meyer A (2006). Sympatric speciation in Nicaraguan crater lake cichlid fish.. Nature.

[10] Savolainen V, Anstett M-C, Lexer C, Hutton I, Clarkson JJ (2006). Sympatric speciation in palms on an oceanic island.. Nature.

[11] Ryan PG, Bloomer P, Moloney CL, Grant TJ, Delport W (2007). Ecological speciation in South Atlantic island finches.. Science.

[12] Hunt DE, David LA, Gevers D, Preheim SP, Alm EJ (2008). Resource partitioning and sympatric differentiation among closely related bacterioplankton.. Science.

[13] Pastene LA, Mutsuo G, Kanda N, Zerbini AN, Kerem D (2007). Radiation and speciation of pelagic organisms during periods of global warming: the case of the common minke whale, *Balaenoptera acutorostrata*.. Mol Ecol.

[14] Niemiller ML, Fitzpatrick BM, Miller BT (2008). Recent divergence with gene flow in Tennessee cave salamanders (Plethodontidae: *Gyrinophilus*) inferred from gene genealogies.. Mol Ecol.

[15] Hey J (2010). The divergence of chimpanzee species and subspecies as revealed in multipopulation isolation-with-migration analyses.. Mol Biol Evol.

[16] Hare MP, Cipriano F, Palumbi SR (2002). Genetic evidence on the demography of speciation in allopatric dolphins species.. Evolution.

[17] Meyer A, Kocher TD, Basasibwaki P, Wilson AC (1990). Monophyletic origin of Lake Victoria cichlid fishes suggested by mitochondrial DNA sequences.. Nature.

[18] Schliewen UK, Tautz D, Pääbo S (1994). Sympatric speciation suggested by monophyly of crater lake cichlids.. Nature.

[19] Ingman M, Kaessmann H, Pääbo S, Gyllensten U (2000). Mitochondrial genome variation and the origin of modern humans.. Nature.

[20] Gubili C, Bilgin R, Kalkan E, Ünsal Karhan S, Jones CS (2011). Antipodean white sharks on a Mediterranean walkabout? Historical dispersal leads to genetic discontinuity and an endangered anomalous population.. Proc R Soc B.

[21] Hoelzel AR, Dahlheim M, Stern SJ (1998). Low genetic variation among killer whales (*Orcinus orca*) in the Eastern North Pacific and genetic differentiation between foraging specialists.. J Heredity.

[22] Hoelzel AR, Hey J, Dahlheim ME, Nicholson C, Burkanov V (2007). Evolution of population structure in a highly social top predator, the killer whale.. Mol Biol Evol.

[23] Morin PA, Archer FI, Foote AD, Vilstrup J, Allen EE (2010). Complete mitochondrial genome phylogeographic analysis of killer whales (*Orcinus orca*) indicates multiple species.. Gen Res.

[24] Foote AD, Vilstrup JT, de Stephanis R, Verborgh P, Abel Nielsen SC (2011). Genetic differentiation among North Atlantic killer whale populations.. Mol Ecol.

[25] Ford JKB, Ellis GM, Balcomb KC (2000). Killer Whales; The Natural History and Genealogy of Orcinus orca in British Columbia and Washington.

[26] Pitman RL, Ensor P (2003). Three forms of killer whales (*Orcinus orca*) in Antarctic waters.. J Cetacean Res Manage.

[27] Foote AD, Newton J, Piertney SB, Willerslev E, Gilbert MTP (2009). Ecological, morphological and genetic divergence of sympatric North Atlantic killer whale populations.. Mol Ecol.

[28] Pitman RL, Durban JW, Greenfelder M, Guinet C, Jorgensen M (2011). Observations of a distinctive morphotype of killer whale (*Orcinus orca*), type D, from subantarctic waters.. Polar Biol.

[29] Hoelzel AR, Natoli A, Dahlheim ME, Olavarria C, Baird RW (2002). Low worldwide genetic diversity in the killer whale (*Orcinus orca*): implications for demographic history.. Proc R Soc B.

[30] Stoneking M (2000). Hypervariable sites in the mtDNA control region are mutational hotspots.. Am J Hum Genet.

[31] Galtier N, Enard D, Radondy Y, Bazin E, Belkhir K (2006). Mutation hot spots in mammalian mitochondrial DNA.. Genome Res.

[32] Rogers AD (2007). Evolution and biodiversity of Antarctic organisms: a molecular perspective.. Phil Trans R Soc B.

[33] Pitman RL, Perryman WL, LeRoi D, Eilers E (2007). A dwarf form of killer whale in Antarctica.. J Mamm.

[34] Foote AD, Morin PA, Durban JW, Pitman RL, Wade P (2011). Positive selection on the killer whale mitogenome.. Biol Lett.

[35] Ford JKB, Ellis GM, Barrett-Lennard LG, Morton AB, Palm RS (1998). Dietary specialization in two sympatric populations of killer whale (*Orcinus orca*) in coastal British Columbia and adjacent waters.. Can J Zool.

[36] Dahlheim ME, Schulman-Janiger A, Black N, Ternullo R, Ellifrit D (2008). Eastern temperate North Pacific offshore killer whales (*Orcinus orca*): Occurrence, movements, and insights into feeding ecology.. Mar Mamm Sci.

[37] Herman DP, Burrows DG, Wade PR, Durban JW, Matkin CO (2005). Feeding ecology of eastern North Pacific killer whales *Orcinus orca* from fatty acid, stable isotope and organochlorine analyses of blubber biopsies.. Mar Ecol Prog Ser.

[38] Saulitis EL, Matkin CO, Barrett-Lennard LG, Heise KA, Ellis GM (2000). Foraging strategies of sympatric killer whale (*Orcinus orca*) populations in Prince William Sound, Alaska.. Mar Mamm Sci.

[39] Ford JKB, Ellis GM, Matkin CO, Wetklo MH, Barrett-Lennard LG (2011). Shark predation and tooth wear in a population of northeastern Pacific killer whales.. Aquat Biol.

[40] Baird RW, Stacey PJ (1988). Variation in saddle patch pigmentation in populations of killer whale (*Orcinus orca*) from British Columbia, Alaska and Washington State.. Can J Zool.

[41] Zerbini AN, Waite JM, Durban JW, LeDuc R, Dahlheim ME (2007). Estimating abundance of killer whales in the nearshore waters of the Gulf of Alaska and Aleutian Islands using line-transect sampling.. Mar Biol.

[42] Vilstrup JT, Ho SYW, Foote AD, Morin PA, Kreb D (2011). Mitogenomic phylogenetic analyses of the Delphinidae with an emphasis on the Globicephalinae.. BMC Evol Biol.

[43] Huelsenbeck JP, Ronquist F (2001). MrBayes: Bayesian inference of phylogeny.. Bioinformatics.

[44] Posada D (2008). jModelTest: Phylogenetic model averaging.. Mol Biol Evol.

[45] Guindon S, Gascuel O (2003). A simple, fast, and accurate algorithm to estimate large phylogenies by maximum likelihood.. Syst Biol.

[46] Shimodaira H (2002). An approximately unbiased test of phylogenetic tree selection.. Syst Biol.

[47] Shimodaira H, Hasegawa M (2001). CONSEL: for assessing the confidence of phylogenetic tree selection.. Bioinformatics.

[48] Lawson Handley LJ, Manica A, Goudet J, Balloux F (2007). Going the distance: human population genetics in a clinal world.. Trends Genet.

[49] Librado P, Rozas J (2009). DnaSP v5: A software for comprehensive analysis of DNA polymorphism data.. Bioinformatics.

[50] Tajima F (1989). Statistical method for testing the neutral mutation hypothesis by DNA polymorphism.. Genetics.

[51] Hey J, Nielsen R (2004). Multilocus methods for estimating population sizes, migration rates and divergence time, with applications to the divergence of *Drosophila pseodoobscura* and *D. persimilis*.. Genetics.

[52] Hey J, Nielsen R (2007). Integration within the Felsentein equation for improved Markov chain Monte Carlo methods in population genetics.. Proc Natl Acad Sci.

[53] Olesiuk PF, Bigg MA, Ellis GM (1990). Life history and population dynamics of resident killer whales (*Orcinus orca*) in the coastal waters of British Columbia and Washington State.. Rep Int Whaling Com Special Issue.

[54] Petit JR, Jouzel J, Raynaud D, Barkov NI, Barnola J-M (1999). Climate and atmospheric history of the past 420,000 years from the Vostok ice core, Antactica.. Nature.

[55] Nei M, Maruyama T, Chakraborty R (1975). The bottleneck effect and genetic variability in populations.. Evolution.

[56] Grant PR, Grant BR (2009). The secondary contact phase of allopatric speciation in Darwin's finches.. Proc Natl Acad Sci USA.

[57] Foote AD, Víkingsson G, Øien N, Bloch D, Davis CG (2007). Distribution and abundance of killer whales in the North East Atlantic SC/59/SM5..

[58] Siengenthaler U, Stocker TF, Monnin E, Lüthi D, Schwander J (2005). Stable carbon cycle-climate relationship during the late Pleistocene.. Science.

[59] Peeters FJC, Acheson R, Brummer G-JA, de Ruiter WPM, Schneider RR (2004). Vigorous exchange between the Indian and Atlantic oceans at the end of the past five glacial periods.. Nature.

[60] Alvarado Bremer J, Vinas J, Mejuto J, Ely B, Pla C (2005). Comparative phylogeography of Atlantic Bluefin tuna and swordfish: the combined effects of vicariance, secondary contact, introgression, and population expansion on the regional phylogenies of two highly migratory pelagic fishes.. Mol Phylogenet Evol.

[61] Schluter D (2009). Evidence for ecological speciation and its alternative.. Science.

