# Supplementary figures and images for: Out of the Pacific and Back Again: Insights into the Matrilineal History of Pacific Killer Whale Ecotypes
Source: PLoS One. 2011 Sep 20;6(9):e24980. doi: 10.1371/journal.pone.0024980 (PMC3176785; doi:10.1371/journal.pone.0024980)

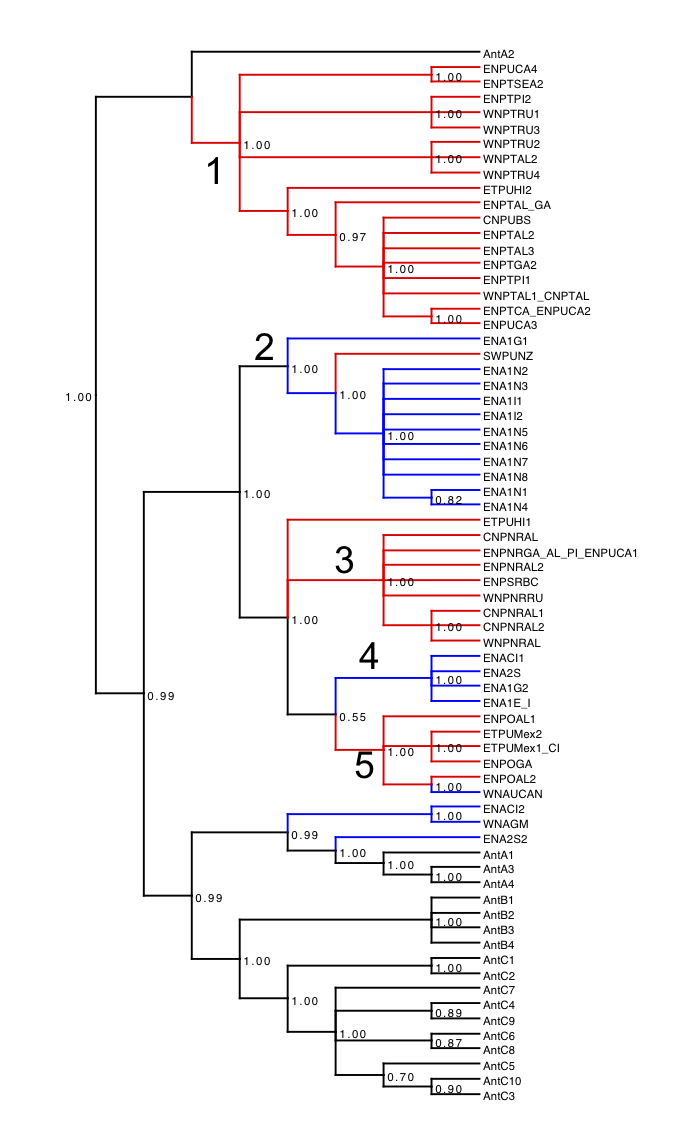

Supplement: Figure S1 — Bayesian phylogeny of the samples analysed showing internal nodes of each clade. Branch colours indicate geographic origin of samples as follows: Antarctic (black), Atlantic (blue) and Pacific (red). Bold numbers indicate the basal node to the clades as used in the AU test and genetic diversity summary statistics. Posterior probabilities are given for nodes of interest. The tree is rooted with long-finned pilot whale (not shown). (TIFF) [file pone.0024980.s001.tiff]
