# Supplementary material for: Out of the Pacific and Back Again: Insights into the Matrilineal History of Pacific Killer Whale Ecotypes
Source: PLoS One. 2011 Sep 20;6(9):e24980. doi: 10.1371/journal.pone.0024980 (PMC3176785; doi:10.1371/journal.pone.0024980)
Supplement: Table S1 — List of GenBank accession numbers of sequences used in this study. (DOC) [file pone.0024980.s002.doc]

| **Haplotype** | **Accession No.** |
| --- | --- |
| AntA1 | GU187217 |
| AntA2 | GU187155 |
| AntA3 | GU187218 |
| AntA4 | GU187219 |
| AntB1 | GU187215 |
| AntB2 | GU187213 |
| AntB3 | GU187212 |
| AntB4 | GU187214 |
| AntC1 | GU187210 |
| AntC10 | GU187209 |
| AntC2 | GU187211 |
| AntC3 | GU187207 |
| AntC4 | GU187205 |
| AntC5 | GU187208 |
| AntC6 | GU187203 |
| AntC7 | GU187153 |
| AntC8 | GU187204 |
| AntC9 | GU187206 |
| CNPNRAL1 | GU187190 |
| CNPNRAL2 | GU187191 |
| CNPUBS | GU187175 |
| CPNRAL | GU187189 |
| ENA1E_I | GU187188 |
| ENA1G1 | GU187176 |
| ENA1I1 | GU187180 |
| ENA1I2 | GU187179 |
| ENA1I2 | GU187183 |
| ENA1N1 | GU187178 |
| ENA1N3 | GU187182 |
| ENA1N4 | GU187177 |
| ENA1N5 | GU187186 |
| ENA1N6 | GU187184 |
| ENA1N7 | GU187185 |
| ENA1N8 | GU187181 |
| ENA2S | GU187154 |
| ENAC1 | HQ405753 |
| ENAC2 | HQ405752 |
| ENAG2 | HQ405755 |
| ENAS2 | HQ405754 |
| ENPNRAL2 | GU187194 |
| ENPNRGA_AL_PI_ENPUCA1 | GU187193 |
| ENPOAL1 | GU187200 |
| ENPOAL2 | GU187201 |
| ENPOGA | GU187197 |
| ENPSRBC | GU187195 |
| ENPTAL2 | GU187173 |
| ENPTAL3 | GU187171 |
| ENPTCA_ENPUCA2 | GU187168 |
| ENPTGA2 | GU187174 |
| ENPTPI1 | GU187169 |
| ENPTPI2 | GU187160 |
| ENPTSEA1 | GU187164 |
| ENPTSEA2 | GU187167 |
| ENPUCA3 | GU187163 |
| ENPUCA4 | GU187199 |
| ETPUHI1 | GU187166 |
| ETPUHI2 | GU187198 |
| ETPUMex1_CI | GU187187 |
| ETPUMex2 | GU187170 |
| SWPUNZ | GU187202 |
| WNAUCAN | GU187216 |
| WNAUGM | GU187159 |
| WNPNRAL | GU187196 |
| WNPNRRU | GU187157 |
| WNPTAL1_CNPTAL | GU187172 |
| WNPTAL2 | GU187161 |
| WNPTRU1 | GU187156 |
| WNPTRU2 | GU187158 |
| WNPTRU3 | GU187192 |
| WNPTRU4 | GU187162 |
